# The intelligent football players’ motion recognition system based on convolutional neural network and big data

**DOI:** 10.1016/j.heliyon.2023.e22316

**Published:** 2023-11-14

**Authors:** Xin Wang, Yingqing Guo

**Affiliations:** a College of Physical Education and Music，Qilu University of Technology (Shandong Academy of Sciences), Jinan, 250353, China; b China Institute of Sport Science, Beijing, 100061, China

**Keywords:** Deep learning, Intelligent image processing, Functional strength training, Convolutional neural network, BiLSTM

## Abstract

This article focuses on evaluating the efficacy of intelligent image processing techniques using deep learning algorithms in the context of football, to present pragmatic solutions for enhancing the functional strength training of football players. The article commences by delving into the prevailing research landscape concerning image recognition in football. It then embarks on a comprehensive examination of the prevailing landscape in soccer image recognition research. Subsequently, a novel soccer image classification model is meticulously crafted through the fusion of Space-Time Graph Neural Network (STGNN) and Bi-directional Long Short-Term Memory (BiLSTM). The devised model introduces the potency of STGNN to extract spatial features from sequences of images, adeptly harnessing spatial information through judiciously integrated graph convolutional layers. These layers are further bolstered by the infusion of graph attention modules and channel attention modules, working in tandem to amplify salient information within distinct channels. Concurrently, the temporal dimension is adroitly addressed by the incorporation of BiLSTM, effectively capturing the temporal dynamics inherent in image sequences. Rigorous simulation analyses are conducted to gauge the prowess of this model. The empirical outcomes resoundingly affirm the potency of the proposed deep hybrid attention network model in the realm of soccer image processing tasks. In the arena of action recognition and classification, this model emerges as a paragon of performance enhancement. Impressively, the model notched an accuracy of 94.34 %, precision of 92.35 %, recall of 90.44 %, and F1-score of 89.22 %. Further scrutiny of the model's image recognition capabilities unveils its proficiency in extracting comprehensive features and maintaining stable recognition performance when applied to football images. Consequently, the football intelligent image processing model based on deep hybrid attention networks, as formulated within this article, attains high recognition accuracy and demonstrates consistent recognition performance. These findings offer invaluable insights for injury prevention and personalized skill enhancement in the training of football players.

## 1 Introduction

In the 21st century, the advancement of science and technology has led to significant improvements in people's living standards. One noteworthy outcome of this technological progress is the widespread acceptance of computer vision, a pivotal field of study. Particularly, the integration of computer vision techniques, such as deep learning, has gained substantial recognition for its capacity to process video images and extract motion-related information without the need for physical contact. This functionality empowers the recognition and analysis of human movements during physical activities. The swift evolution of artificial intelligence (AI) has ushered in a paradigm shift in its integration within the sports arena, particularly within the revered domain of soccer. In tandem, the ascendancy of data analytics and the proliferation of intelligent sports products have cast a spotlight on the alluring prospects presented by the infusion of AI technology into the expanse of sports [1,2]. However, the current landscape depicts a relatively constrained scope of AI applications within football, both in terms of breadth and depth. The majority of football training methods still hinge on coaches' experiential guidance to shape players' skills and performance. This reliance limits the potential for enhancing and standardizing footballers' poses and abilities [3]. Consequently, the application of deep learning and other algorithmic approaches to football training has emerged as a central focus of scientific inquiry within the realm of sports research.

Football, characterized by intense competition, frequent scrambles, and fierce antagonism, places considerable emphasis on players' strength training as an integral facet of physical conditioning. The underlying objective of physical training is to optimize players' physical capabilities and attributes, enabling them to effectively leverage their technical and tactical prowess and, ultimately, elevate their competitive prowess. Diverse scenarios within football pose multifaceted demands on players' speed and strength, underscoring the significance of these attributes [4,5]. Within the context of football, rapid changes in movement direction stand as a critical aspect of players' skill set. Traditionally, coaches often observed players grappling with cognitive agility limitations while swiftly altering their movement trajectories. Remarkably, players' understanding of training methodologies was frequently deemed inadequate, and their recognition of the pivotal role of training remained deficient [6]. Incorrect execution during training sessions could potentially result in player injuries or misconceptions regarding sports methodologies. Hence, the accurate cultivation of players’ functional strength assumes paramount importance. Deep learning, a prominent algorithm within the realm of AI, has garnered significant attention. The adoption of machine learning methodologies for motion recognition investigations has garnered substantial traction within the sports domain. Notably, algorithms have been effectively employed to accurately categorize commonplace activities like standing, walking, running, and reclining, thereby unveiling their distinctive patterns [[7], [8], [9]]. Similarly, deep learning has been applied to recognize human body movements within video sequences, detecting movement speed and direction through the amalgamation of electromyographic signals, acceleration signals, and video data. In more intricate sports such as badminton, table tennis, tennis, and football, data from worn acceleration sensors can be collected and subsequently subjected to identification and classification using deep learning and analogous algorithms [10]. Consequently, deep learning and AI technology hold extensive promise across the sports landscape.

In essence, the objectives encompass mitigating injuries among football players during training while concurrently enhancing their capabilities. These pursuits hold paramount significance in the realm of recognizing and regulating movement within sports. This article brings forth a novel and impactful contribution by introducing a deep hybrid attention network model tailor-made for the processing of football motion images. This model has yielded remarkable enhancements in performance within this specific domain. The core innovation of this paper involves the integration of two advanced components: the Space-Time Graph Neural Network (STGNN) and Bi-directional Long Short-Term Memory (BiLSTM), geared towards football image processing. Furthermore, the integration of attention mechanisms has been adeptly executed to capture pivotal features within the images, resulting in heightened precision in motion recognition and classification tasks. The findings of this study provide valuable experimental evidence that can effectively inform the advancement of the sports sector and contribute to injury prevention strategies within football athlete training.

The article is organized into five distinct sections, each tailored to comprehensively address the research objectives. Section 1, the introduction, provides a background overview, underscores the significance, and highlights the primary contributions of the research. Section 2, the literature review, critically evaluates the current research landscape, identifies unresolved issues, and presents an analysis of recent advancements in the field. Section 3, the research methodology, delineates the employed approach, elaborating on the development of a football motion image recognition model. This model strategically harnesses the strengths of diverse neural networks in image processing to yield optimal outcomes. Section 4, results and discussion, meticulously assesses the performance of the model introduced in the preceding section, conducting thorough comparisons with extant methodologies. This examination yields valuable insights into the model's effectiveness and efficiency. Lastly, Section 5, the conclusion, encapsulates the article's key contributions, offers a comprehensive discourse on research findings, acknowledges study limitations, and proffers suggestions for future research trajectories. This section functions as a succinct denouement of the article, encapsulating core discoveries and accentuating the imperative of continued exploration in the field.

## 2 Literature review

### 2.1 The application trend of deep learning in real-time image processing

The rapid evolution of AI and deep learning has spurred considerable interest in their application within real-time image processing. Bjerge et al. (2022) [11] harnessed computer vision and deep learning to facilitate insect tracking, culminating in the real-time monitoring and tracking of insects via comprehensive image analysis. Kumar et al. (2022) [12] adeptly employed deep learning techniques for face mask detection, employing image processing methodologies to scrutinize facial images within public transportation settings for mask detection purposes. Adem et al. (2023) [13] navigated the realm of sugar beet leaf disease classification through image processing and deep learning, artfully extracting vital features from leaf images to automate the classification of diseases. Choi et al. (2023) [14] embarked on a quantitative analysis of angiogenesis processes within chip images using deep learning-grounded image processing strategies, effectively extracting intricate features to underpin robust quantitative assessments. Ross et al. (2023) [15] harnessed the power of deep learning-based image processing techniques to meticulously process and analyze surface feature images in titanium alloy machining processes, yielding precise measurements and evaluations of surface attributes. Ren (2023) [16] introduced an athlete detection methodology for sports videos anchored in deep learning principles. The research method facilitated accurate athlete identification within sports videos through the adept utilization of deep learning models. The findings, featured in the realm of neural computation and applications, significantly bolstered the analytical capabilities within sports video analysis. In a complementary vein, Meng & Qiao (2023) [17] orchestrated the analysis and design of a dual-feature fusion neural network tailored to sports injury estimation models. The inventive neural network harmonized diverse features, resulting in the precise estimation of sports injuries. This pivotal contribution, showcased within the sphere of neural computation and applications, introduced a novel paradigm for the assessment of sports-related injuries.

### 2.2 Development status of training methods in sports

In sports, proper training poses can significantly enhance players' abilities. Physical fitness stands as a cornerstone among all elements of sports. Strength forms the foundation for speed, endurance, flexibility, and agility. Strong strength capacity guarantees players' mastery of skills, enabling them to execute coaching tactics proficiently. Extensive research has delved into training methods within sports. Mei (2023) [18] introduced a pioneering approach centered on AI-based 3D image analysis to comprehensively examine sports technique features and training methodologies. By scrutinizing athletes’ image data, the research facilitated a profound understanding of sports technique attributes and the intricacies of effective training paradigms. In a parallel stride, Pastel et al. (2023) [19] brought forth compelling evidence regarding the transformative impact of virtual reality (VR) training on the acquisition of complex sports movements. Leveraging the immersive potential of VR technology, the study successfully fostered the acquisition of intricate sports movements, propounding an innovative avenue for training modalities. Le Noury et al. (2022) [20] contributed to the discourse by offering a comprehensive review of the augmented reality (AR) technology landscape and its burgeoning applications in the realm of sports. By encapsulating the contemporary status of AR technology, the authors elucidated its integration within sports and fostered a comprehensive dialogue on its varied potential applications. Concurrently, Dan et al. (2022) [21] ventured into the realm of Internet of Things-based intelligent data aggregation and processing within sports training models. The study demonstrated the potency of IoT technology in orchestrating the intelligent aggregation and processing of data, thus serving as a pivotal support system for enhanced sports training models.

In summary, the studies mentioned above shed light on the conventional nature of training methods in the sports domain. Consequently, there is an increased risk of causing physical injuries to players due to these conventional approaches. Notably, the absence of systematic and intelligent training methodologies is evident. In contrast, the realm of AI, particularly deep learning, holds promise in enhancing the precision of recognizing video sequences and images across various domains. Unfortunately, the incorporation of these technologies in the sports field remains limited. Utilizing deep learning to process and instantly recognize football-related images, along with predicting football speed and direction, offers a promising avenue to enhance the skills of football players. This effort holds significant value for the application of AI in the extensive landscape of sports.

## 3 Methodology

### 3.1 Current status and analysis of functional strength training in football

Stable support holds paramount significance for footballers, with functional core strength emerging as a pivotal factor. This strength empowers the human body to respond adeptly to dynamic demands during exercise. Simultaneously, robust functional core strength equips footballers with the capacity to regulate body acceleration, deceleration, and stability throughout games, thereby enhancing body equilibrium, motor muscle perception, and mitigating the risk of sports-related injuries [22,23]. Indeed, across all sports, various movements intricately hinge upon an exercise chain centered on core muscles. Robust core muscles assume the crucial roles of stabilizing and supporting body postures, motor skills, and distinctive technical maneuvers within sports. In this progression, core muscles are responsible for stabilizing the center of gravity and facilitating the linkage and transmission of force—the central nexus of comprehensive force dynamics. As such, core muscles assume a pivotal function in orchestrating the harmonious integration of upper and lower limb actions [24].

Functional core strength constitutes the Foundational capacity to establish core stability within the human body. This strength transcends its role as a passive stabilizer, extending its influence to actively generate force in the context of football and other competitive sports. This characteristic positions functional core strength as a pivotal “force fountainhead” for various exercises [25]. Footballers confront the recurrent necessity to execute rapid accelerations, abrupt halts, and instantaneous alterations in movement trajectory. These demands necessitate a finely honed capability to swiftly modify motion trajectories and effectuate rapid spatial adjustments in response to opponents or the ball. Unfortunately, the realm of football and other competitive sports' training often entails instances of sports-related injuries or stagnated progression attributed to erroneous training poses. In this context, AI technologies, specifically deep learning, are judiciously employed in tandem with modern functional core strength training methodologies. The objective herein is to explore footballers’ adeptness in modulating speed and strength—an inquiry of considerable consequence that augments the intelligent evolution and enhancement of sports.

### 3.2 Application analysis of deep learning in sports images

Deep learning, a prominent AI algorithm, constitutes a simulated rendition of the intricate neural connectivity found in the human brain. This algorithm adeptly captures the distinctive attributes of images, sounds, and text data by subjecting them to a series of successive transformational stages [26]. In the domain of image processing, several well-established neural network architectures, such as Convolutional Neural Networks (CNN), Recurrent Neural Networks, and Long Short-Term Memory (LSTM) networks, hold notable prevalence.

**Fig. 1:**
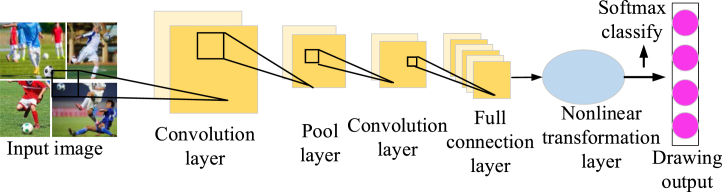
Visual illustration of CNN applied to sports image processing.

CNN, in particular, operates as a feedforward neural network, typically encompassing an amalgamation of diverse layers, including convolutional layers, fully connected layers, and pooling layers [27]. The process of hierarchical feature extraction inherent in CNNs unfolds through successive convolutional layers. This intricate mechanism systematically captures local features from images, evolving from rudimentary to intricate representations. This distinctive property of CNNs is notably advantageous for dissecting intricate attributes such as sports movements and postures embedded within sports images. The profound utility of CNNs in sports image analysis is depicted in Fig. 1.

Usually, CNN will perform convolution operations in multiple dimensions. If a two-dimensional matrix *I* is used as input, then a two-dimensional kernel *K* is used for convolution calculation, as shown in Equation (1):

(1) $$S\left(i,j\right)=\left(I\cdot K\right)\left(i,j\right)=\sum_{m}\sum_{n}I\left(m,n\right)K\left(i-m,j-n\right)$$

in (1), *i, j, m,* and *n* are all fixed parameters, referring to the dimension and order of the matrix. Convolution can be exchanged and can be equivalently written as Equation (2).

(2) $$S\left(i,j\right)=\left(I\cdot K\right)\left(i,j\right)=\sum_{m}\sum_{n}I\left(i-m,j-n\right)K\left(m,n\right)$$

The property of the convolution operation's interchangeability arises due to the inversion of the convolution kernel concerning the input. While the index of the input progresses, the kernel's index regresses. This kernel flipping is solely intended to facilitate interchangeability. Although this property holds significance for theoretical proofs, its application in neural networks is not particularly influential. Notably, certain neural network libraries feature a cross-correlation function that closely resembles the convolution operation, albeit lacking the kernel-flipping aspect, as shown in Equation (3).

(3) $$S\left(i,j\right)=\left(I\cdot K\right)\left(i,j\right)=\sum_{m}\sum_{n}I\left(i+m,j+n\right)K\left(m,n\right)$$

CNNs have proven effective in pixel classification of original sports images, such as those in soccer; however, the dynamic nature of sports images often encompasses temporal variations in movements, as evident in actions like dribbling and shooting in soccer. This article introduces a notable refinement to the CNN paradigm by integrating the STGNN into sports image processing. The STGNN framework adeptly accommodates both spatial features intrinsic to images and the temporal sequences inherent in sports actions, thereby adeptly capturing the temporal nuances of movements. This augmentation culminates in heightened accuracy for sports action analysis and recognition [28,29]. The strategic inclusion of attention mechanisms imbues the model with the ability to dynamically recalibrate node weights, accentuating pivotal information and amplifying the model's adaptability. Consequently, the amalgamation of attention mechanisms yields superior spatio-temporal data analysis and prediction capabilities.

**Fig. 2:**
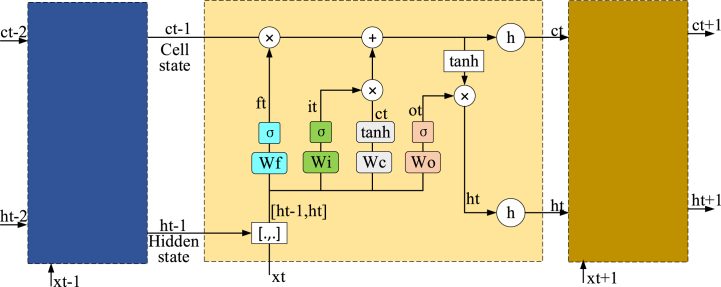
The structural diagram of the LSTM network.

Notably, within the realm of sports image processing, the temporal dimension of actions holds paramount importance. LSTM, a recurrent neural network architecture tailored for sequential data modeling, emerges as a fitting choice. Its inherent ability to capture the intricate temporal relationships inherent in sports actions effectively enhances the comprehension and analysis of dynamic sporting processes. LSTM network includes a cell state, a hidden state, and four gates with different functions: the forget gate *f*_*t*_, the input gate *i*_*t*_, the cell state gate *C*_*t*_, and the output gate *o*_*t*_ [30]. The structure of the LSTM network is displayed in Fig. 2 below.

Within the architecture of the LSTM network, both the cell state and the hidden state serve as repositories for housing internal states. The pivotal role of the forget gate is to ascertain the information that necessitates deletion from the cell state—essentially, the process of “forgetting.” This operation is executed by utilizing the Sigmoid function, as depicted by Equation (4).

(4) $$\left\{ \begin{array}{l} f_{t}=\sigma \left(W_{f}\cdot \left[h_{t-1},x_{t}\right]+b_{f}\right)\\ i_{t}=\sigma \left(W_{i}\cdot \left[h_{t-1},x_{t}\right]+b_{i}\right)\\ {\tilde{C}}_{t}=\tanh\left(W_{C}\cdot \left[h_{t-1},x_{t}\right]+b_{C}\right)\\ C_{t}=f_{t}*C_{t-1}+i_{t}*{\tilde{C}}_{t}\\ o_{t}=\sigma \left(W_{o}\cdot \left[h_{t-1},x_{t}\right]+b_{o}\right)\\ h_{t}=o_{t}*\tanh\left(C_{t}\right) \end{array}\right.$$

Equation (4) features distinct elements: *W*_*f*_ designates the weight matrix affiliated with the output gate; *x*_*t*_ represents the prevailing input; *h*_*t*_ embodies the output from the antecedent step; *b* signifies the bias term; *W*_*C*_ stands for the weight matrix engendered through the cell state; *W*_*o*_ refers to the weight matrix inherent to the output gate.

The integration of BiLSTM introduces a crucial mechanism for bidirectional information propagation. This entails the assimilation of information not only preceding the current time step, as is typical in forward information flow, but also subsequent to the current time step through backward information flow. This bidirectional framework enriches contextual comprehension significantly, affording a more holistic grasp of dependency relationships within sequential data [31]. In the context of sports image processing, the employment of BiLSTM leads to notable advancements in predictive performance.

### 3.3 Construction and analysis of a football image classification model integrating stgnn with bilstm

**Fig. 3:**
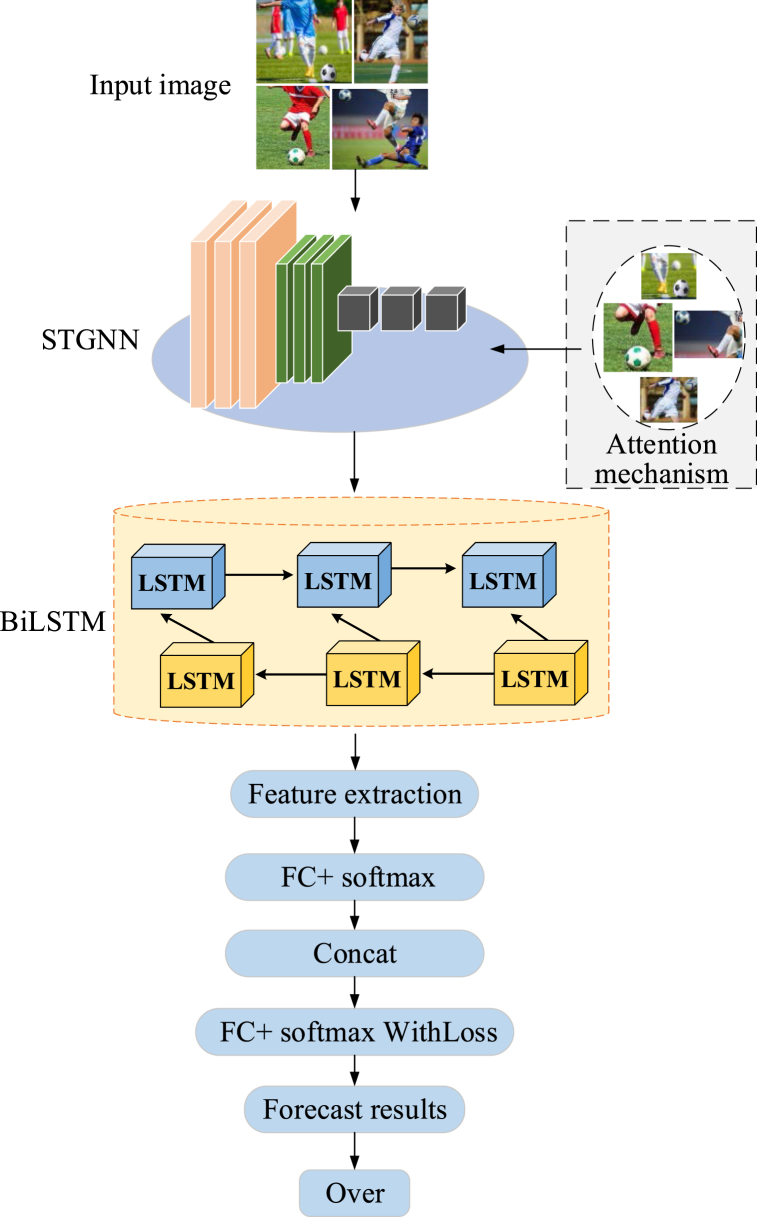
Framework of the football image classification model based on STGNN integrated with BiLSTM.

In this article, the construction and analysis of a football image classification model encompassing the integration of STGNN with BiLSTM are presented. The proposed approach seeks to execute robust feature extraction and classification tasks on football images. Initially, the STGNN is harnessed to extract spatial features from image sequences, bolstered by the integration of attention mechanisms that contribute to the model's augmented generalization capacity. Within this algorithmic paradigm, convolutional and pooling operations operate within the spatial dimension, adeptly capturing intricate motion attributes embedded within the image sequences. Sequentially, the BiLSTM comes into play, orchestrating the sequential modeling of these static image features. A comprehensive feature representation is synthesized through techniques such as concatenation or other fusion methodologies by effectively fusing the spatial features extracted via STGNN and the temporal features encoded by BiLSTM. This enriched feature representation subsequently feeds into task-specific models designed for recognizing football actions and predicting player movements within video images. The schematic depiction of the intricate framework governing the football image classification model realized through the integration of STGNN with BiLSTM, is illustrated in Fig. 3.

As depicted in Fig. 3, this model commences by employing the STGNN to execute convolutional operations within the graph structure's temporal and spatial dimensions. This dual-pronged approach adeptly captures salient features embedded within the image sequence, thereby yielding a comprehensive spatial feature representation. A judiciously integrated attention mechanism comes into play during the extraction of spatial features. This mechanism empowers the model to bestow heightened focus on pivotal features, thereby enhancing its acumen for extracting essential attributes from specific image frames. This attention mechanism operates dynamically, autonomously adjusting weights to enhance the model's emphasis across diverse time steps within the image sequence. Subsequently, having amassed spatial features, these feature sequences seamlessly transition to the BiLSTM network to engender temporal information modeling. BiLSTM excels in capturing temporal relationships via bidirectional flows, thus fostering a richer comprehension and analysis of actions and movements manifested within the image sequence. Following traversal through both STGNN and BiLSTM, an enriched feature representation, amalgamating spatial and temporal facets, is realized. This composite representation is then channeled to the classification layer, culminating in the adept execution of image classification tasks. The strategic fusion of spatial and temporal insights endows the model with heightened expressiveness, thereby enabling finer demarcation of diverse action categories embodied by football players in distinct images.

Notably, the spatial graph convolutional layer within STGNN is fortified with a graph attention module. This augmentation empowers the model to learn network parameters and orchestrates an optimization process on the connectivity graph. The outcome of this optimization endeavor is a more refined graph structure tailored to encapsulate intricate actions, thereby augmenting the model's potency in forecasting football player actions [32]. Specifically, with the integration of the graph attention module, the formulation governing the spatial graph convolutional layer is crystallized, as presented in Equation (5).

(5) $$f_{out}=\sum_{k}^{K}w_{k}\left(f_{in}\left(A_{k}^{\prime }+B_{k}\right)\right)$$

in Equation (5), $A^{\prime }$ corresponds to a data-driven graph matrix, while *B* denotes the graph attention matrix. This matrix serves as a pivotal tool in enhancing the model's ability to meticulously encapsulate actions for each individual sample, thereby elevating the model's personalized performance. For a given input feature $f\left(v_{ti}\right)$, the model resorts to the deployment of two distinct convolutional layers. These layers are meticulously orchestrated to transform the input feature into K-vectors and Q-vectors, as succinctly depicted in Equation (6).

(6) $$\left\{ \begin{array}{l} K_{ti}=W_{K}f\left(v_{ti}\right)\\ Q_{ti}=W_{Q}f\left(v_{ti}\right) \end{array}\right.$$

in Equation (6), $W_{K}$ and $W_{Q}$ symbolize the weight matrices intrinsic to the two distinct convolutional layers. These matrices are directly linked to the *Q*-vector and *K*-vector of the node $v_{ti}$, respectively. Subsequently, the mathematical procedure unfolds by computing the inner product of $Q_{ti}$ and $K_{ti}$, as succinctly portrayed in Equation (7).

(7) $$u_{\left(t,i\right)\to \left(t,j\right)}=\left\langle Q_{ti},K_{tj}\right\rangle$$

In Equation (7), the nodes $v_{ti}$ and $v_{tj}$ are inherently situated within the same temporal phase. The symbol ⟨,⟩ signifies the inner product's representation. Within this construct, the derived inner product $u_{\left(t,i\right)\to \left(t,j\right)}$ serves as a measure of the similarity existing between the nodes $v_{ti}$ and $v_{tj}$. To further refine and standardize the numerical range of u to fall within the boundaries of 0 and 1, the transformative influence of the Softmax function is brought into play. This intricate normalization process is explicitly illustrated through Equation (8).

(8) $$\alpha_{\left(t,i\right)\to \left(t,j\right)}=\frac{\exp\left(u_{\left(t,i\right)\to \left(t,j\right)}\right)}{\sum_{u=1}^{N}\exp\left(u_{\left(t,i\right)\to \left(t,n\right)}\right)}$$

in Equation (8), α represents the normalized similarity of the inner product *u*. Therefore, through the integration of the graph attention module into the spatial graph convolutional layer, the model becomes adept at learning the weights for arbitrary pairs of action types across different movements. This data-driven strategy enriches the model's adaptability, enabling accurate action prediction even in the presence of varied datasets.

For achieving a more sophisticated depiction of motion features, a supplementary attention mechanism was introduced in the channel domain by integrating a channel attention module after the spatial graph convolutional layer [33]. The output $f_{out}\in R^{H\times W\times C}$ of the graph attention module was employed as the input for this module, facilitating a “squeeze” operation to infuse global information. The temporal and spatial dimensions underwent an average pooling process, as illustrated in Equation (9).

(9) $$z_{c}=\frac{1}{H\times W}\sum_{i=1}^{H}\sum_{i=1}^{W}m_{c}\left(i,j\right)$$

here, $m_{c}\in R^{H\times W}$ signifies an element of the matrix *Z*, which is the resultant output following this stage. Further, a transformation is enacted on the output *Z*, as delineated in Equation (10).

(10) $$S=\sigma \left(W_{2}\delta \left(W_{1}Z\right)\right)$$

in Equation (10), *W*_*1*_ and *W*_*2*_ correspond to the two weight matrices affiliated with the fully connected layers, *σ* represents the Sigmoid activation function, and *δ* signifies the Parametric Rectified Linear Unit (PReLu) activation function. The matrix *S* is subject to multiplication with the input feature map *f*_*out*_, and via a residual mechanism, it is compounded with the original input feature map. The outcome of this procedure furnishes the ultimate output of the channel attention module. This intricate interplay ensures that each channel's signal undergoes multiplication by the pertinent learned weights. This approach augments the network's emphasis on pivotal channel-related information, consequently heightening the extraction of spatio-temporal features from football players' movements within the images.

**Fig. 4:**
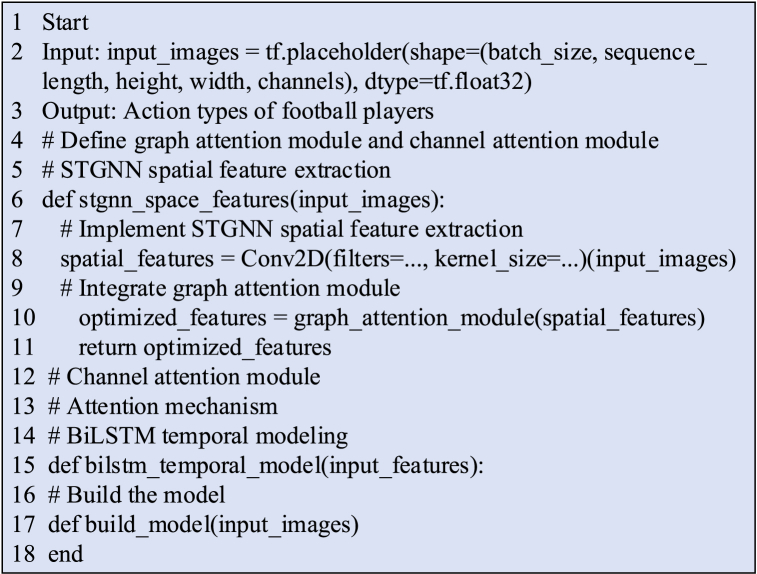
Pseudocode flowchart illustrating the integration of STGNN and BiLSTM Algorithm.

The pseudocode outlining the seamless integration of the STGNN with the BiLSTM algorithm is presented in Fig. 4.

## 4 Results and discussion

### 4.1 Datasets collection and hyperparameters setting

The MATLAB platform is adopted to simulate and analyze the proposed football real-time image processing model based on deep learning. The Federation of Association Football (FIFA) 19 Complete Player Dataset, accessible at (https://www.kaggle.com/datasets/), is identified as the primary source of soccer motion images for this article. Furnished by the International FIFA, this dataset encompasses comprehensive profiles of a myriad of soccer players worldwide. It encapsulates a spectrum of data categories, inclusive of players’ foundational profiles, skill evaluations, attribute specifics, designated positions, and club associations. Each player within the dataset is attributed skill ratings corresponding to diverse soccer proficiencies (e.g., shooting, passing, speed), integral for simulating their in-game performance. Technical and physical attributes of players (e.g., strength, endurance, agility) are meticulously cataloged, as these attributes substantially impact their performance during matches. Intricate annotations elucidate the role and position of each player on the field, such as forward, midfielder, defender, and beyond.

In this article, the image data extracted from the dataset undergo a rigorous preprocessing phase. Image preprocessing aims to render the image data suitable for training and analysis within the models constructed for this article. The specific steps involved in image preprocessing are detailed in Table 1.

**Table 1:** Image preprocessing steps.

Steps	Content
Image loading	The retrieval of image data from the FIFA 19 complete player dataset, where each image represents a football player.
Image desensitization	The removal of sensitive information, such as player names, ages, nationalities, and other personal details.
Image resizing	Adjusting all images to uniform dimensions, typically 224 × 224 pixels, ensures consistency across the dataset.
Image normalization	The scaling of pixel values to a standardized range, typically between 0 and 1.
Data augmentation	The enhancement of data diversity through random operations like rotation, flipping, cropping, etc., aimed at improving model generalization.
Label encoding	The assignment of appropriate categories or labels to each image, such as different football actions or player attributes.

**Fig. 5:**
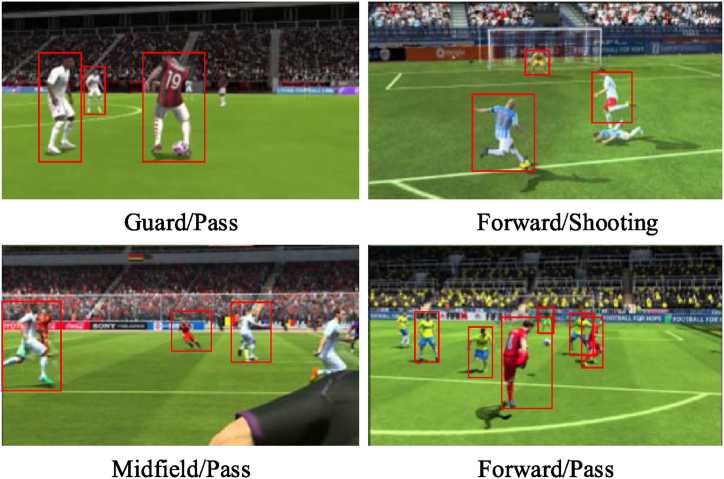
Example images of some football sports in the FIFA 19 Complete Player Dataset.

The rigorous image preprocessing procedures culminate in creating a final dataset comprising a total of 5891 image samples, with each image sample encompassing at least one distinct football action. Fig. 5 presents a partial display of images from this dataset.

**Fig. 6:**
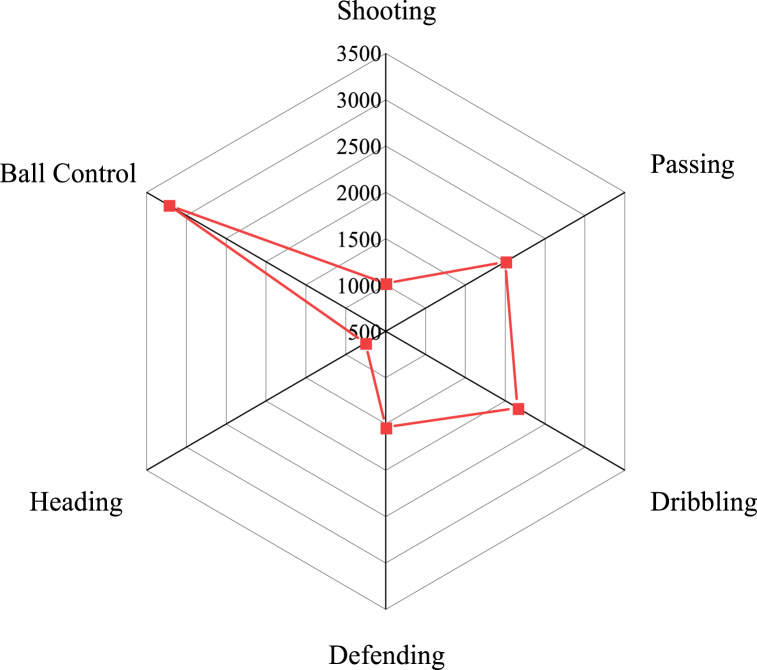
Statistical result of the distribution of different actions in image samples.

As illustrated in Fig. 5, the dataset's meticulous annotations encompass the precise positioning and role of each player on the field, denoting designations such as forward, midfielder, and defender. These annotations offer a comprehensive elucidation of the players' strategic placements and functional roles during matches. Furthermore, the dataset attributes individual players with diverse soccer aptitudes, including but not limited to shooting, passing, and speed. These skill ratings underpin the foundational dataset for simulations and shed light on players' proficiencies and specialized prowess across multifaceted dimensions of the game. Upon conducting a descriptive statistical analysis of the dataset, it becomes evident that it can be categorized into six distinct actions: shooting, passing, dribbling, defending, heading, and ball control. This categorization is visually represented in Fig. 6.

Fig. 6 illustrates the distribution of actions in the dataset. Notably, ball control actions comprise the largest segment, with a total of 3216 instances. These samples holistically encompass essential player data, skill evaluations, attribute particulars, positions, and club affiliations. These selected samples are harnessed for a repertoire of tasks, including action detection, action recognition, and allied analyses. During the simulation phase employing this dataset, the assorted images spanning diverse player positions, attributes, and skills are judiciously partitioned into training and testing sets, employing a ratio of 70 % for training and 30 % for testing. In the context of handling data, particularly in the realm of football games and training data, there may be a presence of sensitive information, including player names, ages, nationalities, and other personally identifiable details. It is imperative to ensure strict adherence to privacy regulations by meticulously anonymizing and safeguarding personal data. Furthermore, the dataset employed in this experiment rigorously complies with ethical standards, thereby ensuring the research's legality and ethical adherence. It is of utmost importance that, when data is no longer required, it is securely destroyed or deleted to forestall any unwarranted accumulation of data.

**Fig. 7:**
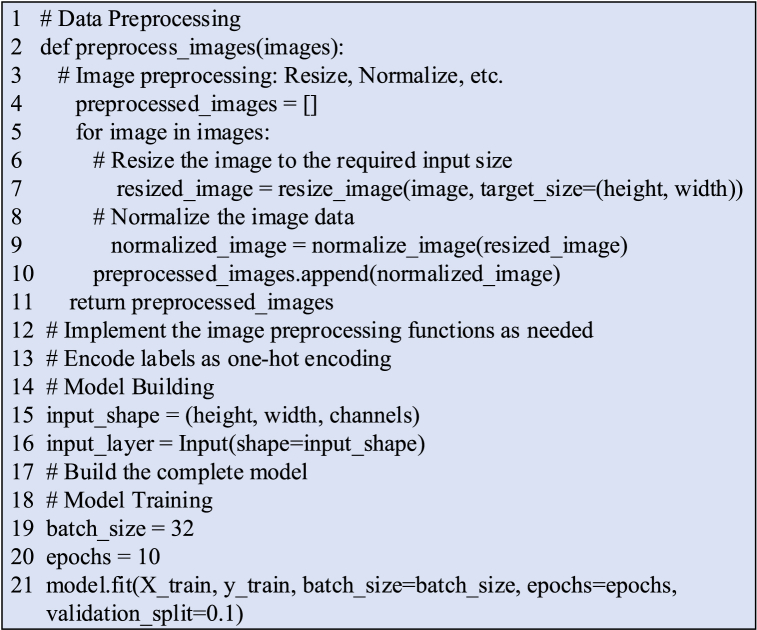
Image data processing and training process pseudo code flow.

Fig. 7 provides the pseudocode flow outlining the processing and training of image data.

The algorithmic model is meticulously crafted using the training dataset, where diverse parameter values are assigned to yield a spectrum of results. Subsequently, the test set serves as a repository to document the network model's outcomes, permitting a comparative analysis against established algorithms. To ensure the robustness of the accuracy comparison experiment, the subsequent hyperparameters are meticulously set: The model in this article undergoes 80 epochs of training, employing a learning rate of 0.002, a batch size of 128, a 1 × 3 convolution kernel size, ReLU activation function, a dropout rate of 0.5 within the CNN framework, and optimization through the Adam optimizer. Furthermore, a comprehensive comparative experimental analysis is conducted to ensure objectivity and rigor. This analysis encompasses the proposed model algorithm and benchmarked it against alternative model algorithms, namely CNN [34], STGNN [35], BiLSTM [36], Ren (2023), and Meng & Qiao (2023). The evaluation of these alternative models was grounded in a range of essential metrics, including accuracy, precision, recall, and the F1 score. All simulation experiments are conducted on a Windows 10 operating system housing a 3.0 GHz processor and 8 GB RAM. The employed CPU is CORE-i7-7700HQ. Table 2 offers a succinct overview of the specific modeling tools harnessed in these empirical undertakings.

**Table 2:** Modeling tools.

	Tool's version
Simulation software	MATLAB
Matrix transportation	Numpy1.12.6; Pandas 0.23.0
Programming language	Python 3.2
Development tool	Tensorflow 1.8.0 and R3.4.2
System	Windows 10
CPU	Intel Core i7-7700@4. 2 GHz 8 cores
GPU	NVIDIA GeForce 1060 8G

The experiment relies on a suite of software and hardware components. MATLAB is employed for executing specific mathematical models and algorithms. Python version 3.2 serves as the primary language for writing and executing the core code for simulation experiments. This article also utilizes essential Python libraries, namely Numpy 1.12.6, which is pivotal for managing large multi-dimensional arrays and matrix data, and Pandas 0.23.0 for data processing and analysis. The synergy of Python, Numpy, and Pandas facilitates efficient data management and analysis. TensorFlow, in combination with Python, plays a significant role in the experiment. Additionally, R, a programming language designed for data analysis and statistical modeling, is integrated into the workflow. For operations executed on a Graphics Processing Unit (GPU), Compute Unified Device Architecture (CUDA) is harnessed. CUDA, an NVIDIA-provided parallel computing platform and application interface, substantially boosts computational speed in tasks involving extensive computations and deep learning model training.

### 4.2 Analysis of image segmentation accuracy across different algorithm models in the training set

**Fig. 8:**
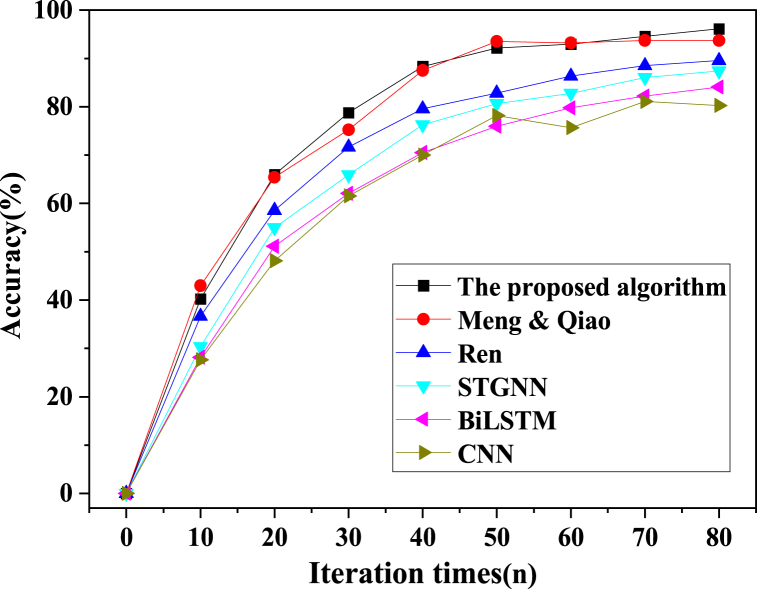
Image processing accuracy results of different models on the training dataset.

**Fig. 9:**
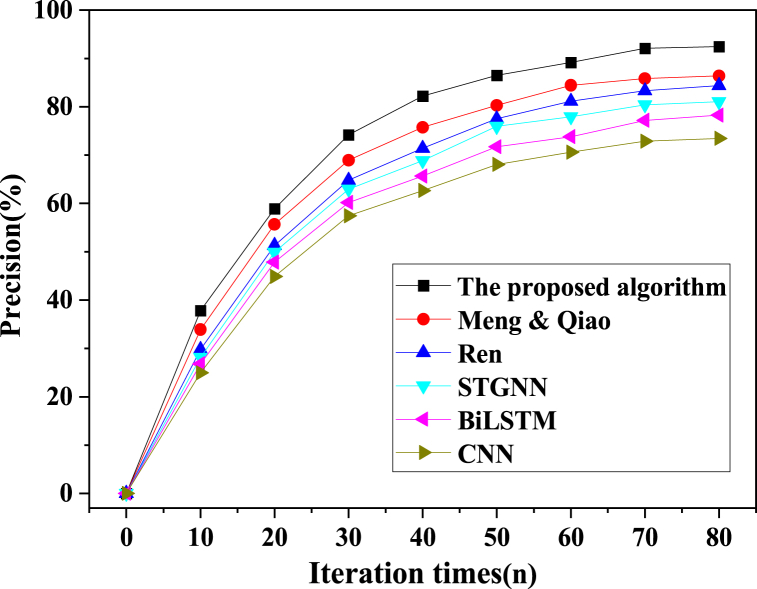
Image processing precision results of different models on the training dataset.

**Fig. 10:**
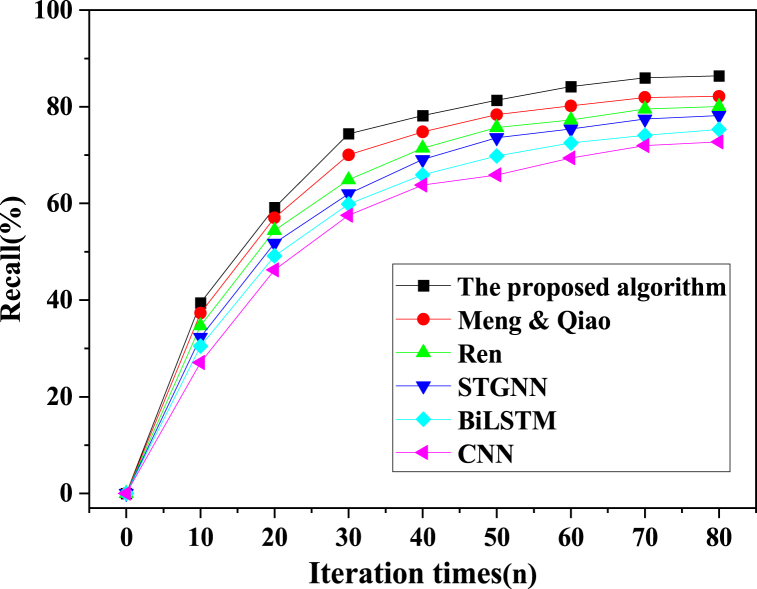
Image processing recall results of different models on the training dataset.

**Fig. 11:**
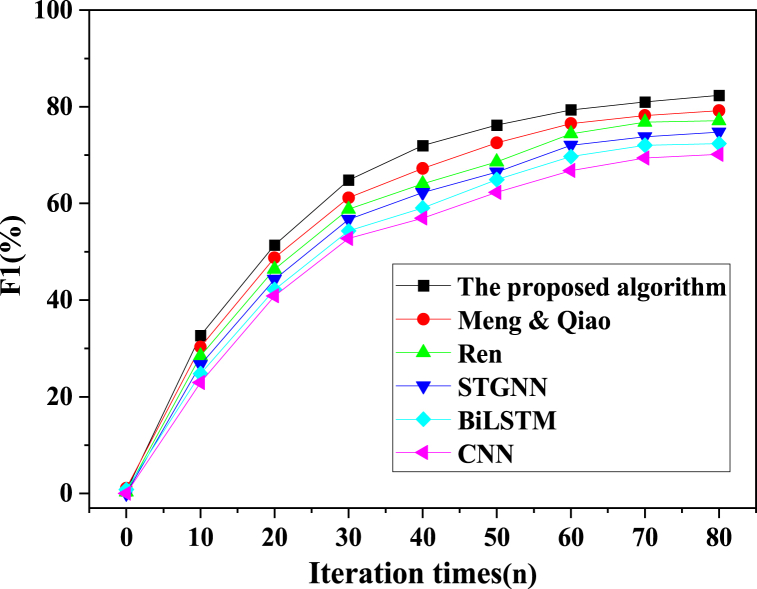
Image processing f1 results of different models on the training dataset.

In the pursuit of a comprehensive evaluation, the proposed model algorithm was meticulously juxtaposed with alternative model algorithms, including CNN, STGNN, BiLSTM, Ren (2023), and Meng & Qiao (2023). This evaluative process hinged on critical metrics such as accuracy, precision, recall, and the F1 score. The outcomes of the training phase are meticulously portrayed in Fig. 8, Fig. 9, Fig. 10, Fig. 11.

As delineated in Fig. 8, Fig. 9, Fig. 10, Fig. 11, a comprehensive evaluation is conducted on the proposed model vis-à-vis other algorithms, encompassing pivotal performance metrics comprising accuracy, precision, recall, and the F1 score. Notably, each model's image segmentation accuracy exhibits a progressive ascent in tandem with iterative cycles, culminating in eventual stabilization. Of particular significance, the current investigation attains an impressive accuracy benchmark of 96.16 %, signifying a notable elevation of no less than 2.46 % over algorithms employed by peers. The hierarchy of classification accuracy, from highest to lowest, distinctly emerges as follows: the algorithm underpinning the model in this study > Meng & Qiao (2023) > Ren (2023) > STGNN > BiLSTM > CNN. Simultaneously, a meticulous traversal through the prism of precision, recall, and the F1 score underscores a gradual yet definitive augmentation in classification accuracy, echoing the iterative progression. These values coalesce into a triumvirate of 92.43 %, 82.38 %, and 86.42 %, respectively. Unmistakably, the algorithmic model expounded in this paper decisively outperforms contemporaneous alternatives. This model's astute fusion of STGNN and BiLSTM emerges as a beacon of enhanced recognition and predictive precision, markedly surmounting deep learning algorithms hitherto employed. In sum, this culminates in the model's remarkable acuity in encapsulating the intricacies of soccer player actions within video images, thereby delivering a heightened degree of accuracy in facilitating athlete action classification and prediction.

### 4.3 Comparative analysis of image recognition across different model algorithms in the test set

**Fig. 12:**
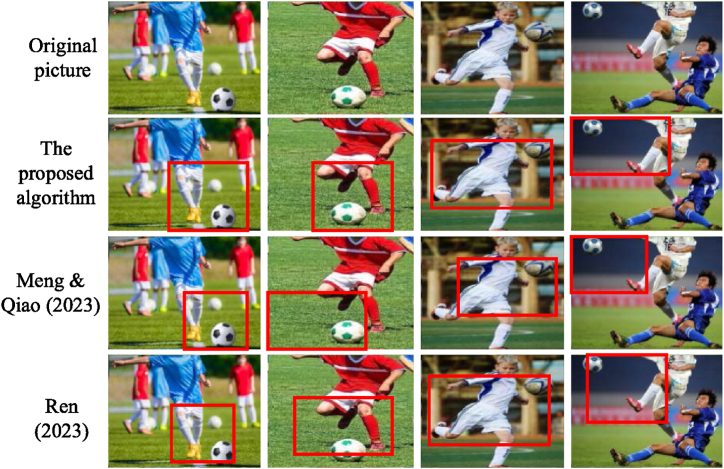
Comparison and analysis of real-time football image processing and recognition effects of different models.

The assessment of accuracy among the model algorithms outlined above distinctly underscores the superiority of the proposed algorithm, leading the pack in performance. Following suit are models advocated by researchers in kindred domains, exemplified by Ren (2023) and Meng & Qiao (2023). Hence, an exhaustive juxtaposition of these three image recognition algorithms is meticulously executed within the test set, culminating in the illustrative portrayal depicted in Fig. 12.

Fig. 12 illustrates the application of the proposed algorithm, Ren's (2023) model, and the model by Meng & Qiao (2023) for the recognition of original images. The algorithm articulated in this paper excels in accurately discerning the contact points between limbs and the football, alongside pinpointing the ball's precise location. The models advanced by Ren (2023) and Meng & Qiao (2023) manifest adeptness in recognizing targets within specific regions. However, due to the potential omission of specific target areas, their feature extraction might fall short of comprehensiveness. Even with subsequent refinement of features from these areas, sustaining robust classification performance poses a challenge. In contrast, in comparison to the model algorithms advocated by Ren (2023) and Meng & Qiao (2023), the proposed algorithm demonstrates near-consistent real-time image processing and recognition outcomes.

**Fig. 13:**
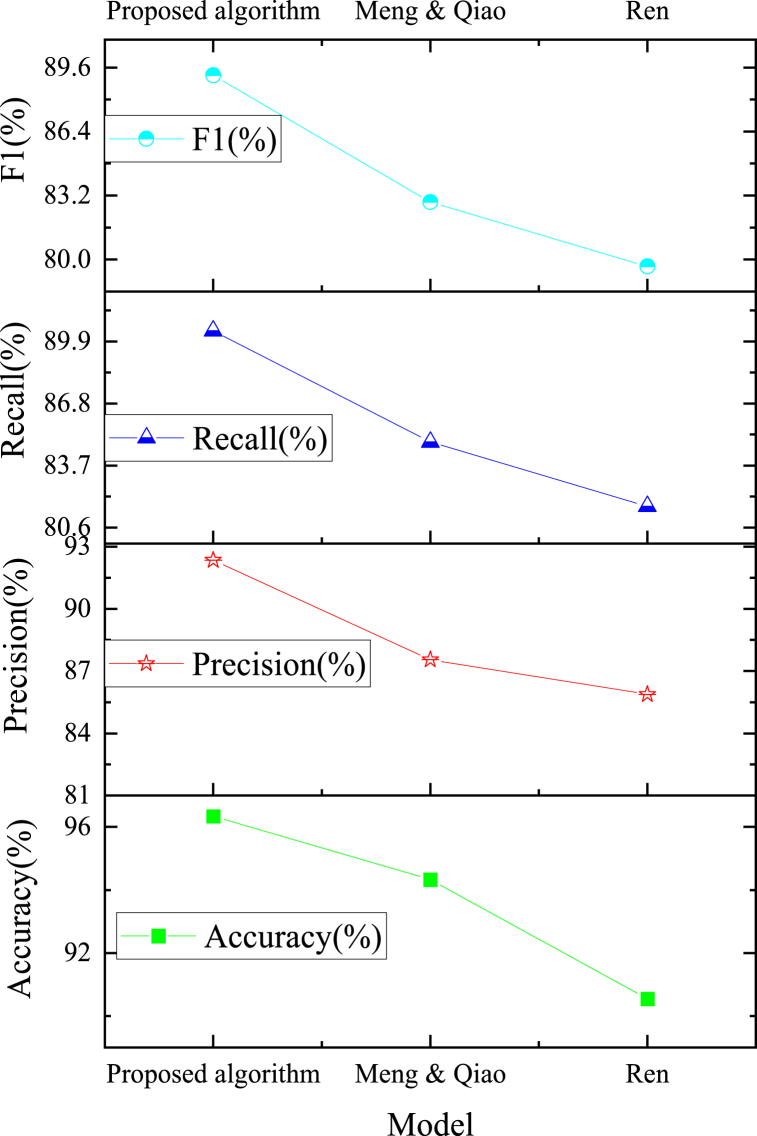
Comparison of recognition performance of different models.

The assessment metrics, namely accuracy, precision, recall, and F1 score, are judiciously chosen to facilitate a comprehensive juxtaposition of recognition performance across the three algorithms employed in football image analysis. The outcomes of this comparative analysis are depicted in Fig. 13.

The outcomes illustrated in Fig. 13 underscore the superiority of the STGNN-fused BiLSTM algorithm, as developed here, for football action image processing in comparison to the models advocated by Ren (2023) and Meng & Qiao (2023) in terms of classification recognition efficacy. Notably, this model achieves an accuracy of 94.34 %, a precision of 92.35 %, a recall of 90.44 %, and an F1 score of 89.22 %. These metrics outperform those of the other two models, showcasing the remarkable accuracy and recognition prowess of the proposed model within the realm of football action image processing.

### 4.4 Discussion

The analysis of image segmentation results reveals a consistent trend across all models. As the number of iteration cycles increases, the image segmentation accuracy steadily improves before eventually stabilizing. Notably, the proposed algorithm stands out by achieving an impressive accuracy rate of 96.16 %. This figure surpasses alternative algorithms by a margin of at least 2.46 %, underscoring the superior classification accuracy of the model presented in this study. This exceptional performance can be attributed to the unique characteristics of the proposed algorithm. By ingeniously combining STGNN and BiLSTM, the algorithm significantly enhances the precision of soccer player action classification and prediction, surpassing the capabilities of previously employed deep learning algorithms. This observation aligns with the findings of Chughtai et al. (2022) and underscores the potential of this model to provide robust support for soccer action recognition.

From the perspective of original image recognition, it becomes evident that the algorithm presented here excels at identifying key points of contact between limbs and the soccer ball, as well as accurately pinpointing the soccer ball's position in the original images. When compared to the algorithms proposed by Ren (2023) and Meng & Qiao (2023), this algorithm distinguishes itself through more comprehensive feature extraction and a more robust classification performance. Furthermore, within the domain of image action processing, the integration of STGNN with BiLSTM demonstrates outstanding performance. This encompasses higher levels of accuracy, precision, recall, and F1 scores. These results underscore the algorithm's remarkable accuracy and recognition capabilities within the realm of soccer action image processing, in alignment with the perspective advanced by An et al. (2023).

In conclusion, the algorithm model presented here demonstrates exceptional performance in the field of soccer action image processing and classification, surpassing other model algorithms. This achievement is paramount for improving the accuracy of soccer player action classification and prediction. The findings of this article offer valuable insights for both scholars and practitioners in the field, serving as a point of reference for future image processing studies.

## 5 Conclusion

### 5.1 Research outcomes

In the current digital era, the continuous maturation of big data and deep learning technologies has brought about profound changes in the fields of computer vision and sports analysis, laying a solid foundation for the development of intelligent soccer player motion recognition systems. Against this backdrop, this article proposes an enhanced deep learning algorithm that successfully develops a smart soccer player motion recognition system based on STGNN and BiLSTM, integrating an attention mechanism. The integration of the attention mechanism results in the development of a soccer image classification model based on STGNN combined with BiLSTM [37,38]. Comprehensive simulations of this model demonstrate its exceptional real-time image processing accuracy during both the training and testing phases of the dataset. It consistently surpasses the performance of other relevant models in the field, achieving an accuracy rate consistently exceeding the threshold of 86.42 %. Moreover, the real-time processing and recognition analysis of soccer images consistently yields reliable recognition results. This empirical evidence supports the enhancement of soccer player training and individual skills in the realm of sports. The model provides a comprehensive understanding of soccer players’ performance in matches, encompassing their movements, positions, and skill levels. This article benefits coaches and team managers and offers valuable feedback to athletes, aiding them in enhancing their technical and tactical proficiency [39].

### 5.2 Research limitations

Despite the significant research outcomes achieved, this article has several limitations. Firstly, this article focuses on recognizing and analyzing the movements in soccer player images, with relatively less emphasis on direct player training. Consequently, this system has not yet fully harnessed its potential to enhance the individual skill levels of soccer players. Secondly, while the experiments in this article demonstrate excellent performance, the model's capabilities are still constrained by data quality and quantity. Enlarging the dataset to a larger scale and greater diversity could further enhance the system's performance and generalization capability [40,41].

### 5.3 Future prospects

In future research, AI technologies can be leveraged to establish a comprehensive cloud-based platform for more extensive soccer data analysis. This platform will include abundant soccer game and training data, which will undergo comprehensive data mining and analysis. The focus will be on balancing technological advancements with practical applications, exploring their profound impacts on the sports domain. Additionally, an in-depth investigation into the technical proficiency of different players in actual events will be conducted to accelerate improving their skills. This strategic development will have far-reaching implications for promoting functional strength training and skill enhancement among soccer players. Ultimately, by continuing to integrate big data, deep learning, and computer vision technologies, it is anticipated that technological advancements will be actively balanced, ushering in more innovation and progress in the soccer field and inspiring research and applications in other domains.

## Data availability statement

Data will be made available on request.

## CRediT authorship contribution statement

**Xin Wang:** Writing – original draft, Methodology, Formal analysis, Data curation. **Yingqing Guo:** Writing – review & editing, Supervision, Software, Resources.

## Declaration of competing interest

The authors declare that they have no known competing financial interests or personal relationships that could have appeared to influence the work reported in this paper.
